# Prevalence and clinical impacts of obstructive sleep apnea in patients with idiopathic pulmonary fibrosis: A single-center, retrospective study

**DOI:** 10.1371/journal.pone.0291195

**Published:** 2023-09-26

**Authors:** Jae Ha Lee, Ji Hoon Jang, Jin Han Park, Sunggun Lee, Ji Yeon Kim, Junghae Ko, So Young Jung, Dae-Wook Kim, SungMin Hong, Hang-Jea Jang

**Affiliations:** 1 Division of Pulmonology and Critical Care Medicine, Department of Internal Medicine, Haeundae Paik Hospital, Inje University College of Medicine, Busan, Republic of Korea; 2 Division of Rheumatology, Department of Internal Medicine, Haeundae Paik Hospital, Inje University College of Medicine, Busan, Republic of Korea; 3 Department of Pathology, Haeundae Paik Hospital, Inje University College of Medicine, Busan, Republic of Korea; 4 Division of Endocrinology, Department of Internal Medicine, Haeundae Paik Hospital, Inje University College of Medicine, Busan, Republic of Korea; 5 Department of Dermatology, Haeundae Paik Hospital, Inje University College of Medicine, Busan, Republic of Korea; 6 Department of Orthopedic Surgery, Haeundae Paik Hospital, Inje University College of Medicine, Busan, Republic of Korea; 7 Division of Pulmonary and Critical Care Medicine, Department of Internal Medicine, Busan Paik Hospital, Inje University College of Medicine, Busan, Republic of Korea; Peking University People’s Hospital, CHINA

## Abstract

**Background:**

Idiopathic pulmonary fibrosis (IPF) is an interstitial lung disease with chronic, progressive lung fibrosis with a poor prognosis. Recent studies have reported a high prevalence of obstructive sleep apnea (OSA) in IPF patients and an association with poor prognosis. This study aimed to evaluate the prevalence, risk factors, and clinical effects on mortality of OSA in patients with IPF.

**Methods:**

Clinical data were retrospectively analyzed in 167 patients with IPF at Haeundae-Paik Hospital, Republic of Korea. A type 4 portable device was used to monitor OSA, and an apnea-hypopnea index of 5 events per sleep hour and above was diagnosed as OSA.

**Results:**

The mean follow-up period and age were 26.9 months and 71.4 years, respectively, with male predominance. OSA was confirmed in 108 patients (64.7%). Mild OSA was the most common (62.1%). Independent risk factors for OSA in the multivariate logistic regression analysis were age (odds ratio [OR] 1.07, 95% confidence interval [CI] 1.02–1.13, p = 0.007), body weight (OR 1.05, 95% CI 1.02–1.09, p = 0.002), and risk based on the Berlin questionnaire (OR 2.76, 95% CI 1.12–6.80, p = 0.028). Shorter six-minute walk distance (6MWD) (hazard ratio [HR] 1.00, 95% CI: 1.00–1.00, p < 0.001), acute exacerbation (AE) (HR 13.83, 95% CI: 5.71–33.47, p < 0.001), and higher percentage of cumulative time with oxygen saturation below 90% in total sleep time (HR 1.08, 95% CI: 1.02–1.14, p = 0.007) were risk factors for mortality in IPF patients in the Cox regression analysis.

**Conclusion:**

Approximately two-thirds of the IPF patients had OSA. Older age, higher body weight, and high risk based on the Berlin questionnaire were independent risk factors for OSA in IPF patients. Shorter 6MWD, experience of AE, and night hypoxemia during sleep were associated with a higher risk of mortality in patients with IPF.

## Introduction

Idiopathic pulmonary fibrosis (IPF) is a typical and common chronic, fibrosing interstitial lung disease (ILD) of unknown cause [1]. IPF is characterized by a highly variable clinical course and poor outcomes with a median survival of 2.5–4 years [2–4]. Currently, antifibrotic therapies for IPF have been accepted by international guidelines and used worldwide as an option for pharmacological treatment [5].

However, IPF patients usually have comorbidities that significantly affect their symptoms and clinical courses. In recent international guidelines of the American Thoracic Society/European Respiratory Society/Japanese Respiratory Society/Latin American Thoracic Association (ATS/ERS/JRS/ALAT), it was recommended that patients be evaluated and treated for comorbidities including obstructive sleep apnea (OSA), pulmonary hypertension, gastroesophageal reflux, and cancer, in addition to receiving antifibrotic therapy [6].

Comorbidities may influence the clinical course of patients with IPF, and identification and adequate treatment can improve clinical symptoms and quality of life, even potential survival [7, 8]. Increasing numbers of studies have demonstrated that OSA is frequently accompanied and associated with poor clinical outcomes in patients with IPF [9, 10]. IPF has been known to be more frequently accompanied by OSA, ranging from 17–88% of IPF cases, than the general population [10–13].

OSA is known to affect over a billion people worldwide, according to recent studies [14, 15]. In addition, previous studies found the negative impact of OSA on cardiovascular disease, diabetes, malignancy, immune system, systemic inflammation, and even in pain response [16]. The main clinical features of OSA are caused by repetitive episodes of upper airway collapse that result in hypoxemia and hypercapnia due to apnea or hypopnea, marked intrathoracic pressure swings, and sleep fragmentation that occurs with arousal due to obstructive respiratory events [17]. In addition, it is known that repetitive obstructive apnea, hypoxemia, and hypercapnia during sleep induce chemoreceptor reflexes and increase sympathetic activity through interaction with baroreflex and pulmonary afferent, which eventually acts as a risk for cardiovascular disease [18]. Recent studies have shown that IPF patients with OSA had a higher risk of cognitive impairment, poor sleep efficiency, poor quality of life, and premature mortality than those without OSA [19–21]. However, the prevalence, predictive factors, and long-term impacts on mortality of OSA in patients with IPF have not been well elucidated. Therefore, this study aimed to evaluate the diagnosis of OSA and its impact on mortality during two years of follow-up in patients with IPF.

## Materials and methods

### Study population

From December 2017 to January 2022, 167 patients with IPF were included in this study. The diagnosis of IPF was confirmed according to international guidelines from the ATS/ERS/JRS/ALAT [5, 22]. In terms of home oxygen use, only patients with mild hypoxemia, who did not require oxygen during sleep, were included. Some of the patients in this study were included in a previous study [23]. This study was approved by the Institutional Review Board of Haeundae-Paik Hospital (approval number: 2022-04-035) and also conducted in accordance with the ethical standards of the Declaration of Helsinki. The requirement for written informed consent was waived because of the retrospective nature of this study.

### Clinical information

Clinical information and survival data of all patients were collected retrospectively from medical records and/or National Health Insurance of Korea records. Spirometry was conducted, and the diffusing capacity of the lung for carbon monoxide (DLco) was measured according to ATS/ERS recommendations [24–26]. The results were expressed as percentages of the normal predicted values. The six-minute walk test (6MWT) was performed in accordance with previously published guidelines [27]. Acute exacerbation (AE) of IPF was defined using the criteria suggested by Collard et al. as acute worsening of dyspnea typically within 30 days, with new bilateral lung infiltration that is not fully explained by heart failure or fluid overloading and no identified extra-parenchymal causes [28]. To determine the disease severity of IPF, total scores of the gender, age, and lung physiology (GAP) model were calculated, and stage classification was completed based on the criteria originally suggested by Ley et al. [29].

### Portable monitor

The diagnosis of OSA was made based on the results of portable monitor device testing (SOMNOcheck micro, type 4, Weinmann Medical Technology, Hamburg, Germany), consisting of the heart rate along with airflow and oxygen saturation measured by nasal cannula and pulse oximetry, respectively. The findings of the portable monitor for OSA were scored by an experienced pulmonologist and a registered polysomnographic technologist with 17-year of experience, following the recommendations in the 2018 American Academy of Sleep Medicine (AASM) manual for the scoring of sleep and associated events [30]. All recording data were taken during hospitalization under the control of a sleep technologist and clinical nurse specialist. OSA was defined as an apnea-hypopnea index (AHI) of more than five events per hour. Apnea was scored when the peak signal excursion of airflow fell by ≥90% of the pre-event baseline, lasting for more than 10 seconds. Hypopnea was scored when there was a drop in the peak signal excursion by ≥30% of the pre-event baseline measured with a nasal pressure sensor for more than 10 seconds in connection with either ≥3% oxygen desaturation or arousal [31, 32]. To detect night hypoxemia, the desaturation index and percentage of cumulative time with oxygen saturation below 90% in total sleep time (T90%) were measured. All data were reviewed and analyzed manually. The severity of OSA was classified as mild (AHI 5–15 per hour), moderate (AHI 15–30 per hour), or severe (AHI > 30 per hour). Since the study subjects were inpatients, a clinical nurse specialist checked the arousal and sleep status of patients, and the total sleep time was recorded accordingly.

### Sleep questionnaires

All patients completed sleep questionnaires, including the STOP-BANG and Berlin questionnaires, before portable monitoring for OSA under the guidance of an experienced nurse specialist. These questionnaires have been widely used to assess the presence of OSA as screening tools in real practice [33, 34]. In the STOP-BANG questionnaire, the total score ranges from 0 to 8, and a score of 0 to 2 can be classified as low risk of OSA, whereas a score of 5 to 8 can be classified as high risk for moderate to severe OSA [33]. The Berlin questionnaire consists of three categories: snoring, daytime sleepiness and fatigue, and hypertension and body mass index (BMI), and includes personal information such as age, gender, height, and weight [34].

### Statistical analysis

The data are presented as frequency and percentage for categorical variables and mean ± standard deviation (SD) for continuous variables. Differences in variables were compared across subgroups with the independent t-test or Mann-Whitney’s U test for continuous variables and the chi-square test or Fisher’s exact test for categorical variables as appropriate. To check if the distribution of a variable was normal, Shapiro-Wilk’s test was used. Univariate and multivariate analyses by logistic regression were performed to identify predictive factors independently related to OSA. Prognostic factors for mortality were analyzed using a Cox proportional hazards model or a Cox proportional hazards model with a time-dependent covariate. The proportional hazards assumption was verified by a statistical test based on the scaled Schoenfeld residuals. Multivariate analyses we applied were performed using the forward selection method. Kaplan-Meier curves and the log-rank test were used to compare survival distribution. All statistical analyses were performed with R software, version 4.1.2 (R Foundation for Statistical Computing), and SPSS Statistics software, version 25.0 (IBM Corp, Armonk, NY, USA), and p values less than 0.05 were considered to indicate statistical significance.

## Results

### Baseline characteristics

This study enrolled 167 patients. Of the total patients, the mean age was 71.4 years old, and most patients (79.0%) were male. Most patients (71.3%) were ever-smokers, and the mean smoking history was 25.7 pack-years. The mean body mass index (BMI) was 24.2 kg/m^2^, and the mean neck circumference was 37.4 cm. The majority of patients showed mild restrictive pulmonary function defect in spirometry with moderate DLco reduction. The mean follow-up period was 26.9 months. The baseline characteristics are summarized in Table 1.

**Table 1:** Baseline characteristics between OSA and no OSA groups.

Variable	Total	OSA	No OSA	*P*-value
Patient, no.(%).	167	108 (64.7%)	59 (36.3%)	
Age, years	71.4±6.9	72.4±6.5	69.6±7.4	0.014
Male, no.(%)	132 (79.0)	89 (82.4)	43 (72.9)	0.148
Ever-smoker	119 (71.3)	77 (71.3)	42 (71.2)	0.988
Smoking, pack-year	28.2±24.0	27.1±22.1	30.1±27.1	0.756
Weight, kg	64.7±11.5	66.7±11.0	61.0±11.6	0.002
BMI, kg/m^2^	24.2±3.4	24.8±3.5	23.2±3.0	0.006
Neck circumference, cm	37.4±3.5	37.9±3.4	36.6±3.6	0.024
Home oxygen use	33 (19.8)	20 (18.5)	13 (22.0)	0.586
CRP, mg/dL	0.9±2.6	0.6±1.0	1.5±4.0	0.279
Arterial oxygen pressure, mmHg	96.6±31.5	95.3±32.1	98.7±30.6	0.448
BNP, pg/mL	748.7±3226.8	997.7±3964.2	280.7±466.7	0.308
Pulmonary function test				
FVC, % predicted	75.0±15.7	75.1±15.8	74.8±15.6	0.885
FEV1, % predicted	85.2±16.5	85.9±16.1	83.9±17.3	0.471
DLco, % predicted	57.8±17.3	58.4±16.9	56.7±17.9	0.537
Six-minute walk test				
Distance, m	410.1±110.1	408.1±106.0	414.0±118.9	0.581
Initial SpO_2_,%	96.0±2.7	96.0±2.8	96.0±2.4	0.710
Lowest SpO_2_,%	89.5±7.5	89.5±7.1	89.5±8.3	0.527
GAP stage				
Stage 1, no.(%)	82 (49.1)	51 (47.2)	31 (52.5)	0.523
Stage 2, no.(%)	74 (44.3)	51 (47.2)	23 (39.0)	
Stage 3, no.(%)	11 (6.6)	6 (5.6)	5 (8.5)	
Underlying disease				
Cardiovascular disease, no.(%)	105 (62.9)	73 (67.6)	32 (54.2)	0.088
Diabetes mellitus, no.(%)	63 (37.7)	45 (41.7)	18 (30.5)	0.155
Chronic kidney disease, no.(%)	5 (3.0)	4 (3.7)	1 (1.7)	0.656
Neurovascular disease, no.(%)	25 (15.0)	20 (18.5)	5 (8.5)	0.082
GERD, no.(%)	14 (8.4)	9 (8.3)	5 (8.5)	1.000
Malignancy, no.(%)	34 (20.4)	20 (18.5)	14 (23.7)	0.424
PFD use, no.(%)	156 (93.4)	101 (93.5)	55 (93.2)	1.000
AE, no.(%)	49 (29.3)	28 (25.9)	21 (35.6)	0.190
Death, no.(%)	40 (24.0)	25 (23.1)	15 (25.4)	0.742

Data are presented as mean ± standard deviation or number (%)

OSA, obstructive sleep apnea; BMI, body mass index; CRP, C-reactive protein; BNP, Brain type natriuretic peptide; FVC, forced vital capacity; FEV1, forced expiratory volume in one second; DLco, diffusing capacity of the lungs for carbon monoxide; SpO_2,_ percutaneous oxygen saturation; GAP, gender, age, and lung physiology; GERD, Gastroesophageal reflux disease; PFD, Pirfenidone; AE, Acute exacerbation

108 patients (64.7%) were diagnosed with OSA. The OSA group was older than the non-OSA group and had a higher body weight and BMI, along with a larger neck circumference than the non-OSA group. There were no significant differences in the pulmonary function test, 6WMT, and severity of IPF between the two groups.

### Sleep data and questionnaire

Among the 108 patients with OSA, the mean AHI index was 15.6. Mild OSA was the most common (62.1%), followed by moderate (23.1%) and severe (14.8%) OSA (Table 2). Hypoxemia during sleep, including the mean and lowest values of percutaneous oxygen saturation (SpO_2_), was significantly more severe, with a higher T90% in the OSA group than the non-OSA group. The total number of arousals per hour of sleep was significantly higher in the OSA group.

**Table 2:** Details of sleep data and questionnaire between OSA and no OSA groups.

Variable	OSA	No OSA	*P*-value
Patient, no. (%)	108	59	
AHI index, /hour	15.6±11.0	2.4±1.3	< .001
Mean SpO_2_,%	94.5±1.7	95.2±1.4	0.012
Lowest SpO_2_, %	81.6±7.3	85.8±5.8	< .001
T90%^†^, %	2.2±4.9	1.0±3.6	0.009
Snoring, %	4.7±8.3	3.4±6.4	0.855
Longest apnea, second	36.5±13.1	22.2±11.3	< .001
Arousal index‡, /hour	10.7±8.4	7.6±5.8	0.026
SBQ			
Low risk, no.(%)	22 (20.8)	19 (32.2)	0.041
Moderate risk, no.(%)	60 (56.6)	35 (59.3)	
High risk, no.(%)	24 (22.6)	5 (8.5)	
BQ risk, no.(%)	30 (28.3)	8 (13.6)	0.031

Data are presented as mean ± standard deviation or number (%).

†T90—percentage of cumulative time with oxygen saturation below 90% in total sleep time

‡Arousal index—total number of arousals per hour of sleep

OSA, obstructive sleep apnea; AHI, Apnea-hypopnea index; SpO_2,_ percutaneous oxygen saturation; SBQ, stop bang questionnaire; BQ, berlin questionnaire

According to the STOP-BANG questionnaire, patients belonging to the moderate-risk group for OSA were the most common (56.9%), followed by low-risk (24.8%) and high-risk (17.6%). Based on the Berlin questionnaire, 38 patients (22.8%) were in the high-risk group for OSA, and the high-risk group was significantly more common in the OSA group compared to the non-OSA group (p = 0.031).

### Risk factors for OSA

As a result of unadjusted logistic regression analysis, older age, higher body weight, higher BMI, larger neck circumference, high risk in the STOP-BANG questionnaire, and high risk in the Berlin questionnaire were statistically significant predictive factors associated with OSA in IPF patients (Table 3). In the multivariate analysis, independent risk factors for OSA were older age (odds ratio [OR] 1.07, 95% confidence interval [CI]: 1.02–1.13, p = 0.007), higher body weight (OR 1.05, 95% CI: 1.02–1.09, p = 0.002), and high risk in the Berlin questionnaire (OR 2.76, 95% CI: 1.12–6.80, p = 0.028).

**Table 3:** Risk factors for OSA in IPF patients analyzed by the logistic regression model.

Variable	Unadjusted analysis		Multivariate analysis	
	OR (95% CI)	*P-*value	OR (95% CI)	*P-*value
Age	1.06 (1.01–1.11)	0.016	1.07 (1.02–1.13)	0.007
Male	1.74 (0.82–3.72)	0.151		
Smoking	0.99 (0.98–1.01)	0.443		
Weight	1.05 (1.02–1.08)	0.003	1.05 (1.02–1.09)	0.002
BMI	1.17 (1.05–1.30)	0.004		
Neck circumference	1.11 (1.01–1.23)	0.026		
CRP	0.83 (0.68–1.02)	0.071		
Arterial oxygen pressure	1.00 (0.99–1.01)	0.552		
Pulmonary function				
FVC	1.00 (0.98–1.02)	0.885		
FEV1	1.01 (0.99–1.03)	0.468		
DLco	1.01 (0.99–1.03)	0.535		
Six-minute walk test				
Distance	1.00 (1.00–1.00)	0.753		
Initial SpO_2_	1.00 (0.88–1.14)	0.983		
Lowest SpO_2_	1.00 (0.96–1.05)	0.970		
Difference	1.00 (0.95–1.06)	0.970		
GAP stage				
Stage 1	Reference			
Stage 2	1.35 (0.69–2.62)	0.379		
Stage 3	0.73 (0.21–2.59)	0.626		
Underlying disease				
Cardiovascular disease	1.76 (0.92–3.38)	0.089		
Diabetes mellitus	1.63 (0.83–3.19)	0.157		
Chronic kidney disease	2.25 (0.25–20.63)	0.472		
Neurovascular disease	2.45 (0.87–6.92)	0.090		
GERD	0.98 (0.31–3.08)	0.975		
Malignancy	0.73 (0.34–1.58)	0.425		
SBQ				
Low risk	Reference			
Moderate risk	1.48 (0.70–3.11)	0.300		
High risk	4.15 (1.32–12.99)	0.015		
BQ risk	2.52 (1.07–5.93)	0.035	2.76 (1.12–6.80)	0.028

Data are presented as mean ± standard deviation or number (%).

OSA, obstructive sleep apnea; IPF, Idiopathic pulmonary fibrosis; BMI, body mass index; CRP, C-reactive protein; FVC, forced vital capacity; FEV1, forced expiratory volume in one second; DLco, diffusing capacity of the lungs for carbon monoxide; SpO_2,_ percutaneous oxygen saturation; GAP, gender, age, and lung physiology; GERD, Gastroesophageal reflux disease; SBQ, stop bang questionnaire; BQ, berlin questionnaire

### Survival analysis

**Fig 1:**
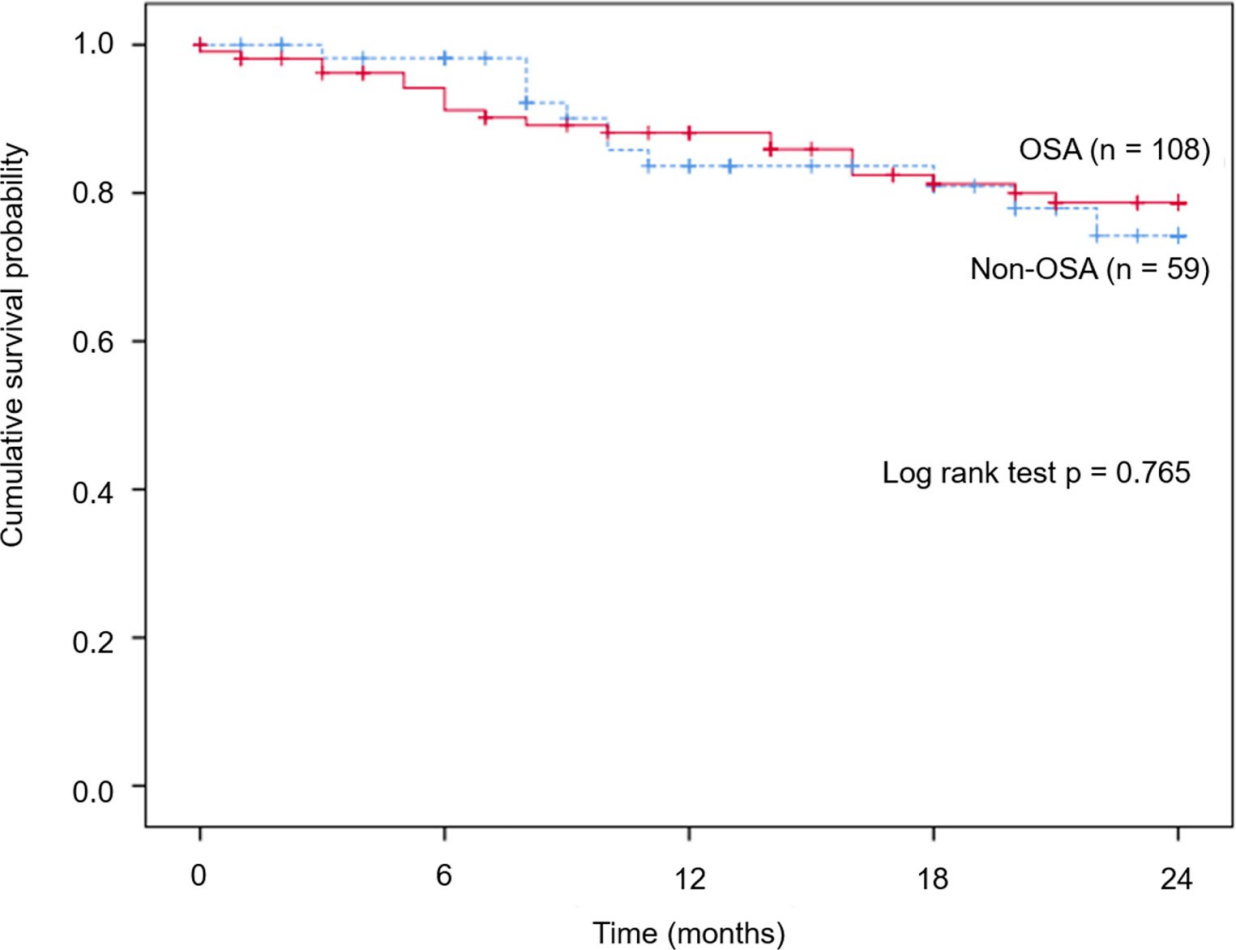
The survival analysis with a cumulative survival probability of OSA and Non-OSA patients during follow-up periods. OSA, obstructive sleep apnea.

During the two-year follow-up period, 37 patients (22.2%) died. The one-year and two-year survival rates for all patients were 86.5% and 77.5%, respectively (Fig 1). There was no significant difference between the two groups in the 1-year and 2-year survival rates. The most common cause of death was acute exacerbation of IPF (77.4%).

In the multivariable Cox analysis, shorter distance during the 6MWT (hazard ratio [HR] 0.99, 95% CI: 0.99–1.00, p < 0.001), acute exacerbation of IPF (HR 13.83, 95% CI: 5.71–33.47, p < 0.001), and higher T90% (HR 1.08, 95% CI: 1.02–1.14, p = 0.007) were significant prognostic factors for mortality in IPF patients. However, the diagnosis of OSA per se was not an independent risk factor for mortality (Table 4).

**Table 4:** Prognostic factors for mortality in patients with IPF by Cox proportional hazards model.

Variable	Unadjusted analysis		Multivariate analysis	
	HR (95% CI)	*P-*value	HR (95% CI)	*P-*value
Age	1.04 (1.00–1.08)	0.055		
Male	0.97 (0.94–1.00)	0.036		
Smoking	0.99 (0.98–1.00)	0.217		
Weight	1.00 (1.00–1.00)	0.000		
BMI	0.99 (0.99–1.00)	0.001		
Neck circumference	0.99 (0.99–1.00)	0.022		
CRP	1.05 (0.99–1.12)	0.111		
Arterial oxygen pressure	0.99 (0.98–1.01)	0.295		
Pulmonary function test				
FVC	0.96 (0.94–0.98)	< .001		
FEV1	1.00 (1.00–1.00)	0.922		
DLco	0.96 (0.95–0.98)	< .001		
Six-minute walk test				
Distance	0.99 (0.99–1.00)	< .001	0.99 (0.99–1.00)	< .001
Initial SpO_2_	0.84 (0.76–0.92)	< .001		
Lowest SpO_2_	0.93 (0.89–0.96)	< .001		
GAP stage				
Stage 1	Reference			
Stage 2	1.00 (0.97–1.02)	0.829		
Stage 3	1.06 (1.02–1.10)	0.005		
Underlying disease				
Cardiovascular disease	1.32 (0.70–2.48)	0.393		
Diabetes mellitus	1.99 (1.10–3.60)	0.023		
Neurovascular disease	2.39 (1.25–4.57)	0.008		
GERD	1.27 (0.45–3.57)	0.649		
Malignancy	1.82 (0.95–3.48)	0.071		
SBQ risk				
Low risk	Reference			
Moderate risk	1.12 (0.54–2.33)	0.759		
High risk	1.08 (0.43–2.74)	0.874		
BQ risk	1.54 (0.80–2.94)	0.195		
AE	10.37 (5.10–21.05)	< .001	13.83 (5.71–33.47)	< .001
PFD use	2.05 (0.28–14.94)	0.477		
OSA	0.85 (0.45–1.59)	0.609		
Lowest SpO_2_	0.98 (0.95–1.01)	0.127		
T90%‡	1.04 (1.00–1.09)	0.037	1.08 (1.02–1.14)	0.007
Longest apnea	0.99 (0.97–1.02)	0.608		

Data are presented as mean ± standard deviation or number (%).

‡T90 –percentage of cumulative time with oxygen saturation below 90% in total sleep time

IPF, Idiopathic pulmonary fibrosis; BMI, body mass index; CRP, C-reactive protein; FVC, forced vital capacity; FEV1, forced expiratory volume in one second; DLco, diffusing capacity of the lungs for carbon monoxide; SpO_2,_ percutaneous oxygen saturation; GAP, gender, age, and lung physiology; GERD, Gastroesophageal reflux disease; SBQ, stop bang questionnaire; BQ, berlin questionnaire; AE, Acute exacerbation; PFD, Pirfenidone; OSA, obstructive sleep apnea

## Discussion

In this study, the prevalence of OSA in patients with IPF was 64.7%, and mild OSA was the most common. Older age, higher body weight, and high risk based on the Berlin questionnaire, were independent factors for OSA. During a two-year follow-up period, the one-year and two-year survival rates were 86.8% and 77.5%, respectively. A shorter distance during the 6MWT, acute exacerbation, and longer duration of night hypoxemia, expressed as a higher T90%, were the significant prognostic factors for mortality; however, diagnosis of OSA itself was not.

About two-thirds of IPF patients were diagnosed with OSA, and mild OSA was the most common. The prevalence of OSA in this study is much higher than that in the general population, even considering the median age of 72.4 years among the enrolled patients. Recently, the prevalence of OSA in the general population was reported to range from 9% to 38% and increased among men and those in advanced age groups [35]. Previous studies support our results. In a prospective cross-sectional study of 30 patients with stable IPF, Sarkar et al. reported that the prevalence of OSA was 56.6% in all patients, and mild to moderate OSA was predominant (76.5%) [21]. Also, Bosi et al., in 34 IPF patients performing full-night polysomnography, showed that 73.5% of patients with IPF were diagnosed with OSA [36]. Although a high prevalence of OSA is observed in patients with IPF, the actual mechanism and pathogenesis are still not well understood. There are some hypotheses, including a bidirectional relationship between IPF and OSA. On the one hand, the decreased lung volumes in restrictive lung diseases such as IPF can reduce upper airway stability and increase resistance due to reducing traction in the upper airway, resulting in sleep disorders, including OSA [37, 38]. On the other hand, OSA is characterized by intermittent hypoxemia for oxidative stress and might cause systemic inflammation and tissue damage, resulting in pulmonary fibrosis [39, 40]. Therefore, it might be necessary to screen for OSA in patients with IPF in terms of comorbidities care and to conduct follow-up exams about IPF in patients with OSA.

As a result of the analysis, older age, higher weight, and high risk based on the Berlin questionnaire were significant risk factors for OSA in patients with IPF. Older age and obesity or high BMI were reported as common risk factors for OSA in the general population as well as in patients with IPF. Previous results also support those findings [10, 41]. Lancaster et al. enrolled 50 patients with IPF and reported that BMI correlated positively with OSA (r = 0.30; p = 0.05) [10]. The Berlin questionnaire is a commonly used screening tool for OSA in the general population [42]. In our previous study, which sought to evaluate the prevalence and predictive factors of OSA in 86 ILD patients (57 with IPF), the Berlin questionnaire was not a significant predictive factor for OSA in patients with ILD [23]. However, in this study which consisted of only IPF patients, the Berlin questionnaire as a screening tool effectively predicted OSA. Considering the practical difficulties in performing polysomnography due to the time and economic cost, it would be helpful to use the Berlin questionnaire as a screening tool in patients with IPF.

In our study, diagnosis of OSA was not a significant risk factor for mortality. This result conflicted with the results of previous studies that OSA acted as a significant factor in mortality in IPF patients [43, 44]. We suggest that the contradictory results may be due to the relatively normal BMI (mean value: 24.2 kg/m^2^) of IPF patients in this study. However, shorter distance during the 6MWT, the experience of AE, and higher T90% were shown to be correlated with disease-related mortality in patients with IPF, similar to the results of a study recently reported by Kolilekas et al. [45]. In a prospective, single-cohort study of 31 patients with IPF, the lowest category of SpO2 was correlated directly with survival (HR 0.897, 95% CI: 0.827–0.972, p = 0.009), but AHI was not significant. Hypoxemia that occurs in situations such as prolonged sleep-related hypoxemia has been known to be associated with poor prognoses, including high mortality and poor quality of life, in all patients with IPF and OSA [46]. Hypoxemia during sleep, known as intermittent hypoxemia, is an important manifestation of OSA due to the nature of the disease, which includes obstruction and increased upper airway resistance. Intermittent hypoxemia induces oxidative stress and inflammation resulting in OSA-related comorbidities [47]. In terms of molecular mechanisms that might be shared by both OSA and IPF pathogeneses, endoplasmic reticulum (ER) stress is known to play a significant role. In a recent in vivo mice model, Shi et al. reported that ER stress-induced lung apoptosis and fibrosis were observed in the lung of mice exposed to intermittent hypoxemia [48]. The aggregated hypoxemia during sleep in patients with IPF might cause a negative impact by impairment of DLco, pulmonary vasoconstriction with ventilation/perfusion mismatching, and progression of IPF [49–51]. Therefore, it is warranted to screen for OSA in patients with IPF and to confirm the existence and severity of night hypoxemia as well as the diagnosis of OSA.

There are some limitations to this study. First, our study was a retrospective observational study with a relatively small number of patients in a single center, which might call into question the generalizability of our findings. However, the baseline characteristics of our patients and some results were similar to those of patients in previous reports [13, 52]. Second, sleep monitoring was performed using a type 4 device instead of a full type 1, gold standard polysomnography test. However, in addition to full type 1 devices, there have been studies that have evaluated the prevalence and severity of OSA through portable devices such as peripheral arterial tonometry alone [53–55]. Nonetheless, our type 4 device has been widely used in clinical practice and validated for diagnosing OSA in previous studies [56]. Third, we did not obtain more data about the adverse impact on patients with IPF, except mortality. Other impacts of OSA, including cognitive impairment, daily symptoms, and quality of life, should not be overlooked. Therefore, a subsequent larger-scale study is needed to evaluate the actual and diverse impact of OSA on patients with IPF. Fourth, the observational period was restricted. Therefore, there are limitations in determining the long-term effects of OSA on survival in IPF patients with the results of this study.

## Conclusion

In conclusion, about two-thirds of patients with IPF had OSA. Older age, higher body weight, and high risk based on the Berlin questionnaire indicated a high risk for OSA. Shorter distance during the 6MWT, experiencing AE, and night hypoxemia during sleep were independent prognostic factors for mortality in patients with IPF.
